# Peroxiredoxin 6 in skin carcinogenesis

**DOI:** 10.18632/oncoscience.41

**Published:** 2014-05-21

**Authors:** Frank Rolfs, Matthias Schäfer, Sabine Werner

**Affiliations:** Department of Biology, Institute of Molecular Health Sciences, ETH Zurich, Switzerland

Non-melanoma skin cancer represents a serious and globally increasing health problem. Therefore, a detailed knowledge on the mechanisms of skin cancer formation is required to develop innovative, efficient and cost-effective strategies for skin cancer prevention and treatment. A major risk factor for cancer formation is oxidative stress, which results from excessive levels of reactive oxygen species (ROS). This can cause oxidative damage of lipids, proteins and DNA, which enhances the risk of mutations [1].

A major enzyme involved in the detoxification of ROS is peroxiredoxin 6 (Prdx6). As a “moonlighting” enzyme it bears two distinct catalytic activities: a cytosolic, selenium-independent peroxidase activity and a lysosomal, calcium-independent phospholipase A_2_ (PLA_2_) activity [2]. Acting as PLA_2_ in lipid catabolism at low pH, Prdx6 hydrolyses the sn-2 ester linkage of phospholipids to release a fatty acid and a lysophospholipid. As a peroxidase, Prdx6 can reduce H_2_O_2_, short chain organic hydroperoxides and phospholipid hydroperoxides at cytosolic, neutral pH [2]. Due to these functions it was of particular interest to determine the role of Prdx6 in skin carcinogenesis. For this purpose we made use of transgenic mice overexpressing Prdx6 in the epidermis as well as of *Prdx6* knockout mice. Interestingly, overexpression of Prdx6 protected keratinocytes of the skin from oxidative damage *in vitro* and *in vivo* [3]. *Vice versa*, mice lacking Prdx6 were more susceptible to oxidative damage in the skin [4]. Based on these data, we analyzed the function of Prdx6 in skin tumorigenesis using genetic and chemically-induced skin cancer models [5]. In the genetic model we used transgenic mice, which express the oncogenes of the human papilloma virus type 8 (HPV8) in keratinocytes and which develop spontaneous skin papillomas within a few weeks. These mice were mated with either *Prdx6* transgenic mice or *Prdx6* knockout mice. Interestingly, loss of Prdx6 enhanced tumor multiplicity in the HPV8 model, while overexpression of Prdx6 significantly reduced the tumor number. Results obtained with *Prdx6* transgenic mice subjected to a model where tumors are induced with a mutagen (DMBA) and a tumor promoter (TPA) confirmed the tumor-preventive effect of this enzyme. In both tumor models, Prdx6 activation had no influence on keratinocyte proliferation, apoptosis or the inflammatory response of the mice. However, following TPA treatment of wild-type mice, we detected an increase in the levels of several epidermal phospholipid hydroperoxides. This increase was much less pronounced in *Prdx6* transgenic mice, but more pronounced in *Prdx6* knockout mice. This finding suggests that Prdx6 reduces oxidative stress and thus protects from lipid peroxidation and most likely also from oxidative DNA damage.

**Figure 1:**
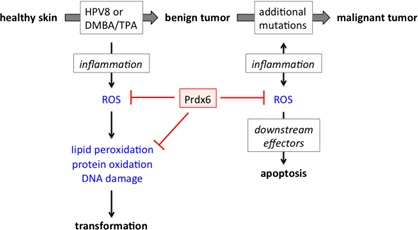
The dual role of Prdx6 in skin carcinogenesis

On the other hand, we found an increase in tumor progression in Prdx6 overexpressing mice, most likely due to protection of tumor cells from inflammation-induced ROS damage and subsequent cell death. Therefore, our data revealed a surprising dual function of Prdx6 in skin tumorigenesis depending on the stage of tumor development: While it protects normal cells from oxidative damage and thus reduces mutagenesis and tumorigenesis, it is also beneficial for cancer cells, thus promoting their survival under stress conditions and subsequent tumor progression. These results are likely to be of human relevance, since we detected strong PRDX6 expression in normal skin and also in cutaneous SCCs [5]. Surprisingly, however, the expression levels were highly variable between different patients. Therefore, it will be important to determine if individuals with strong PRDX6 expression in normal skin are less susceptible to tumor development and *vice versa,* and if PRDX6 overexpression in skin tumors correlates with tumor aggressiveness. These results will provide the basis for the potential use of PRDX6 activators for skin tumor prevention, whereas PRDX6 inhibitors might have therapeutic potential for the treatment of malignant epithelial skin cancers. Consistent with this assumption, thiacremonone, a compound isolated from garlic, was shown to bind to the catalytic site Cys47 of Prdx6 and it reduced the tumor volume of lung carcinoma cells allografted to wild-type mice and in particular of cells allografted to Prdx6 transgenic mice [6]. This promising result points to the usefulness of PRDX6 inhibitors in cancer therapy. These data combined with our results obtained in the skin also demonstrate that Prdx6 activation/ overexpression and ROS-detoxifying strategies in general are not always beneficial. This is also reflected by the recently described acceleration of lung cancer progression in mice by antioxidants [7]. Therefore, activation of ROS-detoxifying enzymes and reduction of ROS levels should be carefully evaluated, in particular in patients with diagnosed cancer.

## References

[1] Hallliwell B (2007). Biochem J.

[2] Fisher AB (2011). Antioxid Redox Signal.

[3] Kümin A (2006). Am J Pathol.

[4] Kümin A (2007). J Cell Biol.

[5] Rolfs F (2013). Cancer Res.

[6] Jo M (2014). PLOS ONE.

[7] Sayin VI (2014). Sci Transl Med.

